# Low Expression of RILPL2 Predicts Poor Prognosis and Correlates With Immune Infiltration in Endometrial Carcinoma

**DOI:** 10.3389/fmolb.2021.670893

**Published:** 2021-05-19

**Authors:** Jinhui Liu, Mengting Xu, Zhipeng Wu, Yan Yang, Shuning Yuan, Jianqiang Liang, Hongjun Zhu

**Affiliations:** ^1^Department of Gynecology, The First Affiliated Hospital of Nanjing Medical University, Nanjing, China; ^2^Department of Urology, The Affiliated Sir Run Run Hospital of Nanjing Medical University, Nanjing, China; ^3^Department of Diagnosis, The first Clinical Medical College of Nanjing Medical University, Nanjing, China; ^4^Department of Oncology, Nantong Third People’s Hospital Affiliated to Nantong University, Nantong, China

**Keywords:** RILPL2, endometrial carcinoma, prognosis, immune infiltration, biomaker

## Abstract

Increasing numbers of biomarkers have been identified in various cancers. However, biomarkers associated with endometrial carcinoma (EC) remain largely to be explored. In the current research, we downloaded the RNA-seq data and corresponding clinicopathological features from the Cancer Genome Atlas (TCGA) database. We conducted an expression analysis, which resulted in RILPL2 as a novel diagnostic biomarker in EC. The dysregulation of RILPL2 in EC was also validated in multiple datasets. The correlations between clinical features and RILPL2 expression were assessed by logistic regression analysis. Then, Kaplan-Meier analysis, univariate and multivariate Cox regression analysis were performed to estimate prognostic values of RILPL2 in the TCGA cohort, which revealed that increased level of RILPL2 was remarkably associated with better prognosis and could act as an independent prognostic biomarker in patients with EC. Moreover, correlation analysis of RILPL2 and tumor-infiltrating immune cells (TIICs) indicated that RILPL2 might play a critical role in regulating immune cell infiltration in EC and is related to immune response. Besides, high methylation level was a significant cause of low RILPL2 expression in EC. Subsequently, weighted gene co-expression network analysis (WGCNA) and enrichment analysis were conducted to explore the RILPL2-involved underlying oncogenic mechanisms, and the results indicated that RILPL2 mainly regulated cell cycle. In conclusion, our findings provided evidence that downregulation of RILPL2 in EC is an indicator of adverse prognosis and RILPL2 may act as a promising target for the therapeutics of EC.

## Introduction

Endometrial carcinoma (EC) is the fourth most common malignant tumors among females in the world behind breast cancer, lung cancer, and colorectal cancer (Sorosky, 2012), with increasing incidence and morbidity over the past years (Siegel et al., 2019). To differentiate the prognosis and its clinical course, The experts divided EC into type I and type II. Type I EC is characterized by estrogen-dependent pathogenesis, low-grade lesions and better clinical outcome, while type II EC is regarded as non-estrogen-dependent, poorly differentiated and has poorer prognosis (Bokhman, 1983). Although most ECs are generally early-stage and low-grade with favorable prognosis, the high-grade group accounts for a disproportionate number of EC deaths (Creasman et al., 2004). Despite EC still occurs more commonly in senior women, it also is being diagnosed in younger women (Moore and Brewer, 2017). Therefore, it is urgent to explore potential indicators that are directly associated with diagnosis in the early stage, therapy, prognosis after treatment and detection of asymptomatic recurrence in endometrial carcinoma.

Rab interacting lysosomal protein like 2 (RILPL2) is a protein-coding gene, encoding a protein that contains a Rab-interacting lysosomal protein-like domain similar to that of Rab-interacting lysosomal protein (RILP). RILP is a Rab7/Rab34 effector which interacts with dynein-dynactin, in order to directly regulate the movement of late endosomes and lysosomes along with microtubules (Johansson et al., 2007). Unlike RILP, RILPL2 does not consist of a Rab7 junction region, which prevents its ectopic expression from the lysosomal compartment (Wang et al., 2004). Although RILPL2 does not affect lysosomal trafficking, it may still act as a functional Rab effector due to its interaction with activated Rab34 and Rab36 (Fukuda et al., 2008; Matsui et al., 2012). The analysis of data obtained from online databases suggested that RILPL2 expressed lower in breast cancer tissues than that in adjacent tissues, and that low RILPL2 expression was prominently positively associated with TNM stage and differentiated grade (Chen et al., 2019). Furthermore, previous studies indicated that RILPL2 functioned in viral replication and could be employed as a potential target for the therapy of Hepatitis C virus (HCV) (Dolan et al., 2013). However, the correlation between RILPL2 and the clinical features of EC has not been studied.

In this research, we first analyze the expression level of RILPL2 and summarize its prognostic roles, biological features, and correlation with tumor immune infiltration in EC patients, mainly according to the public data obtained from the Cancer Genome Atlas (TCGA) database. Summarily, our study suggests that RILPL2 may be a novel predictive indicator for diagnosis, prognosis and promising therapeutic target for EC.

## Materials and Methods

### Collection of the TCGA and the GEO Data

Transcriptome RNA-seq data of 575 samples (containing 23 normal samples and 552 tumor samples) and the corresponding clinical characteristics were downloaded from the TCGA database^1^, which acted as a public repository that includes high-throughput microarray experimental data (Liu et al., 2018). EC sequencing data were generated by the Illumina HiSeq RNA-Seq platform. Then, we conducted pre-processing of clinicopathologic data to eliminate cases with missing or defective information. As a result, the clinical information of 524 patients were reserved for the following analysis.

The GSE17025 dataset was obtained from the Gene Expression Omnibus (GEO) database^2^ (Day et al., 2011), which was used to validate the expression of RILPL2. The GSE17025 dataset contained high-throughput data of 91 EC samples, and 12 atrophic endometrium samples from postmenopausal women.

### Collection of EC Specimens

To validate the aberration of RILPL2 in EC tissues compared to normal tissues, we further collected 15 EC tissues and paired adjacent tissues in the Third People’s Hospital of Nantong in 2020. Ethical approval for the study was granted by the Clinical Research Ethics Committee, the Third People’s Hospital of Nantong, and the current study was performed in conform to the Declaration of Helsinki.

### Total RNA Extraction and Quantitative Real-Time PCR Analysis

To extract total RNA from EC tissues, TRIzol reagent (Thermo Fisher Scientific) was applied. Next, Agilent Bioanalyzer 2100 (Agilent Technologies) with RNA 6000 Nano kit was used to assess the integrity of extracted total RNA. All procedures of real-time quantitative RT-PCR (qRT-PCR) are described as previously (Liu et al., 2020a). Primer sequences for qRT-PCR were presented as follows: RILPL2: ACGTGTATGACATCTCCTACCTG (forward), ACGCGGACGACTTTGAACTG (reverse); GAPDH: CGCT CTCTGCTCCTCCTGTT (forward), CATGGGTGGAATCAT ATTGG (reverse).

### Validation of RILPL2 Protein Level

Human Protein Atlas (HPA), an integration of multi-omics, puts insight into characterizing all human proteins (Uhlen et al., 2015). In this study, the level of RILPL2 in human endometrium and EC samples were compared after acquiring data from the HPA database. Besides, to further validate the protein level of RILPL2, the CPTAC analysis in UALCAN was applied (Chandrashekar et al., 2017).

### Analysis of RILPL2 Expression in EC Samples With Various Clinicopathologic Features

For the data downloaded from the TCGA database, we utilized “ggplot” package in R language (v. 3.6.2) to analyze the RNA-seq data and corresponding clinical features. The relevance between the expression of RILPL2 and various clinicopathologic characteristics was evaluated and emerged by boxplots. The difference of variables was tested using the Wilcoxon signed-rank test or Kruskal-Wallis test. A logistic regression analysis of RILPL2 level and other clinical features was performed *via* R language. *P* < 0.05 was considered as statistically significant.

### Survival Analysis Dependent on RILPL2 Expression

Definitions and outcomes of overall survival (OS), disease-free survival (DFS), and disease-specific survival (DSS) of the TCGA cohort were obtained from the TCGA-Clinical Data Resource (CDR) Outcome. Kaplan-Meier curves were exhibited through the “survival” and “survminer” package in R, *P* < 0.05 was considered to be statistically significant. Univariable and multivariable Cox regression analysis were performed orderly to screen out the prognosis-related factors among the available clinical features, together with the RILPL2 expression profile via the “coxph function” in the “survival” package.

### Correlation Analysis of RILPL2 and Tumor-Infiltrating Immune Cells

ESTIMATE, a method which utilizes the gene expression profiles to speculate the infiltrations of stromal and immune cells in tumor tissues (Yoshihara et al., 2013), was applied to assess the level of immune cell content (immune score), the stromal cell infiltration (stromal score), the stromal-immune comprehensive score (ESTIMATE score) and the tumor purity in each EC sample.

The TIMER tool, an online platform^3^ for comprehensively analyzing the molecular features of tumor-infiltrating immune cells (Li et al., 2017), was applied to study and visualize the correlations between the expression of RILPL2 and the proportion of six tumor-infiltrating immune cells (TIICs), including B cells, CD8 + T cells, CD4 + T cells, macrophages, dendritic cells (DCs) and neutrophils.

CIBERSORT^4^, an analytical tool, was applied to precisely assess the infiltration level of TIICs according to the transcriptome data (Newman et al., 2015). According to the infiltration level of 22 immune cell subtypes reported by the CIBERSORT algorithm, the “vioplot” package was applied to visualize the difference of 22 TIICs in EC samples which detected high and low RILPL2 expression.

### Methylation and Mutation Analysis

On the basis of DNA methylation data downloaded from the TCGA database, we assessed the difference of methylation level of RILPL2 by comparing EC tissues with normal tissues. Furthermore, we analyzed the correlation between RILPL2 methylation and its expression level in EC. Besides, the genetic alterations of *RILPL2* gene in the TCGA dataset were analyzed by cBioPortal tool (Gao et al., 2013).

### WGCNA Analysis

First, as previously described, differentially expressed genes (DEGs) between EC and normal specimens in the TCGA dataset were screened out using the “limma” package with threshold of adjusted *P*-value < 0.05 and | log2FC| > 1 (Liu et al., 2020b). Next, the expression profile of these DEGs was obtained to establish a gene co-expression network by the weighted gene co-expression network analysis (WGCNA) package in R language as previously mentioned (Chen et al., 2020). To match the correlations with the nature regulation relationship, we built a scale-free topology by emphasizing the strong correlations and attenuating the weak correlations with the soft threshold power of β = 3 (scale free *R*^2^ = 0.85). After that, the topological overlap matrix (TOM) was calculated on the basis of adjacency matrices. Finally, we applied the dynamic tree cut algorithm to classify genes according to their expression patterns and merged? gene modules. Gene significance (GS) was deemed as the correlation coefficient between transcriptome expression and module traits. The module eigengene (ME) was calculated as a summary profile for all genes in a module. Module significance (MS) was defined as the correlation coefficient between the module and traits. Module membership (MM) was defined by the correlation coefficient of the module eigengene and transcriptome data. When a gene has MM > 0.80, it was supposed to be the modules’ representative gene, which might possess potential crucial functions.

### Enrichment and PPI Analysis

Gene Ontology (GO) and Kyoto Encyclopedia of Genes and Genomes (KEGG) enrichment analyses were applied to uncover the potential mechanisms of these genes in the turquoise module using DAVID tool^5^. Besides, the key genes were imported into the STRING database^6^ to construct the protein-protein interaction (PPI) network of gene encoding proteins. We set the minimum interaction score as 0.4 (medium confidence).

## Results

### Analysis RILPL2 Expression in EC Patients

**FIGURE 1:**
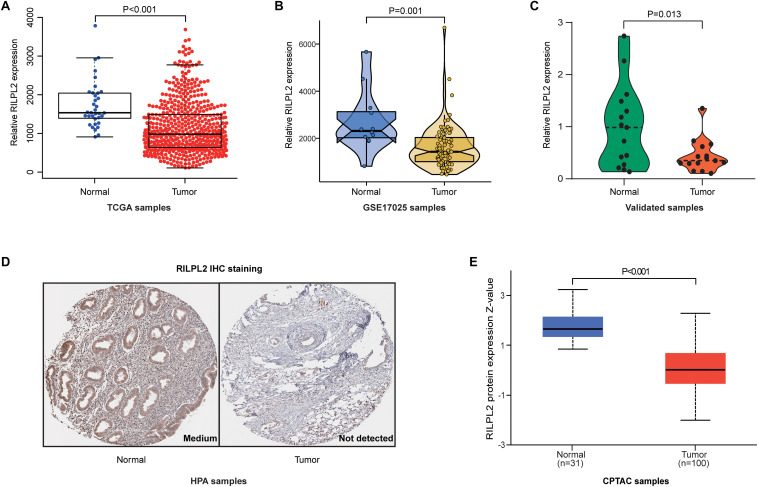
Expression analysis of RILPL2 in EC. **(A–C)** Differential expression of RILPL2 in tumor and adjacent samples from the TCGA dataset, the GSE17025 dataset, and our recruited cohort. **(D)** Representative images shows the samples stained with RILPL2 from the HPA database. **(E)** Differential expression of RILPL2 protein in tumor and adjacent samples from the CPTAC dataset.

By analyzing the data from TCGA, the expression of RILPL2 in the para-cancerous samples was remarkably higher than that in the tumor specimens (*P* < 0.001, Figure 1A). Based on GSE17025 dataset, we observed that RILPL2 also decreased in EC samples compared with para-cancerous samples (*P* < 0.001, Figure 1B). In addition, we further validated RILPL2 expression using our recruited cohort, and the result showed that RILPL2 in tumor samples was obviously lower than that of normal control (*P* = 0.013, Figure 1C). Moreover, the level of RILPL2 protein in human endometrium and EC specimens was compared by calculating the data obtained from the HPA database, and the result showed that RILPL2 protein level was lower in EC samples (Figure 1D). To further validate the protein level of RILPL2, the CPTAC analysis in UALCAN was applied. Not surprisingly, the result validated the downregulated RILPL2 in EC samples again (*P* < 0.001, Figure 1E).

### Association Between RILPL2 Expression and Clinicopathologic Features

**FIGURE 2:**
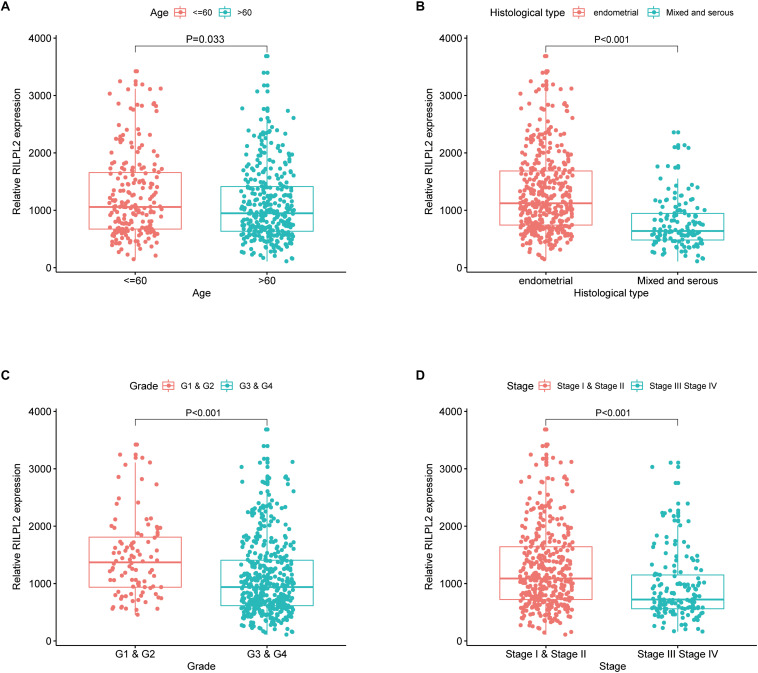
Correlation of RILPL2 expression of and clinicopathologic variables. **(A)** age, **(B)** histological type, **(C)** grade, and **(D)** stage.

A correlation analysis was performed between RILPL2 expression and corresponding clinical characteristics. As presented in Figure 2, decreased expression of RILPL2 is remarkably related to multiple factors, including age (*P* = 0.033, Figure 2A), tumor histological type (*P* < 0.001, Figure 2B), grade (*P* < 0.001, Figure 2C) and clinical stage (*P* < 0.001, Figure 2D). Moreover, as exhibited in Table 1, logistic regression analysis utilizing the median of RILPL2 expression as a classification of the dependent variable indicated that reduced RILPL2 expression was significantly correlated with high grade (Grade 2 vs. Grade 1, *P* = 0.005; Grade 3 vs. Grade 1, *P* < 0.001; Grade 4 vs. Grade 1, *P* < 0.001), histological type (Mix vs. Endometrial, *P* = 0.042; Serous vs. Endometrial, *P* < 0.001) and pathological stage (Stage III vs. Stage I, *P* < 0.001; Stage IV vs. Stage I, *P* < 0.001).

**TABLE 1:** Logistic regression analysis between RILPL2 expression and clinical features.

**Clinical characteristics**		**Odds ratio**	***P* value**
Age	≤60 vs. >60	0.99 (0.98–1.01)	0.483
Grade	2 vs. 1	0.42 (0.23–0.77)	0.005
	3 vs. 1	0.17 (0.10–0.28)	<0.001
	4 vs. 1	0.06 (0.01–0.26)	<0.001
Histology	Endometrial vs. mix	0.40 (0.16–0.95)	0.042
	Endometrial vs. serous	0.40 (0.10–0.28)	<0.001
Stage	II vs. I	0.57 (0.31–1.03)	0.064
	III vs. I	0.34 (0.22–0.53)	<0.001
	IV vs. I	0.22 (0.09–0.51)	<0.001

### Prognostic Value of RILPL2 in EC Patients and Cox Regression Analysis

**FIGURE 3:**
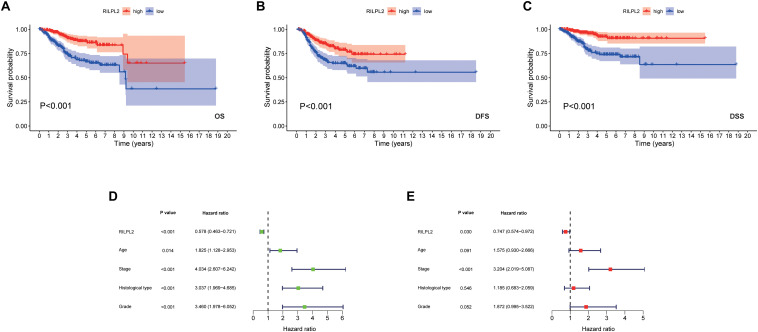
Prognostic value of RILPL2 in EC patients in the TCGA database. **(A)** OS, overall survival. **(B)** DFS, disease free survival. **(C)** DSS, disease specific survival. **(D)** univariate analysis of RILPL2. **(E)** multivariate analysis of RILPL2.

Based on the fact of RILPL2 downregulation in EC, we further focused on its prognostic role in patients with EC. Kaplan-Meier analysis of the TCGA dataset demonstrated that the patients with high RILPL2 expression led to significantly better OS (*P* < 0.001) DFS (*P* < 0.001), and DSS (*P* < 0.001) in EC patients compared to the low RILPL2 expression group (Figures 3A–C). Univariate Cox analysis revealed that RILPL2 (HR = 0.578, 95%CI: 0.463–0.721, *P* < 0.001) were low-risk factor, while age, stage, histological type, and grade were high-risk factors (Figure 3D). Furthermore, multivariate Cox analysis showed that RILPL2 (HR = 0.747, 95%CI: 0.574–0.972, *P* = 0.030) was independently related to OS (Figure 3E), which implied that RILPL2 could be an independent prognostic predictor for EC.

### The Relevance of RILPL2 Expression With Immune Infiltration

**FIGURE 4:**
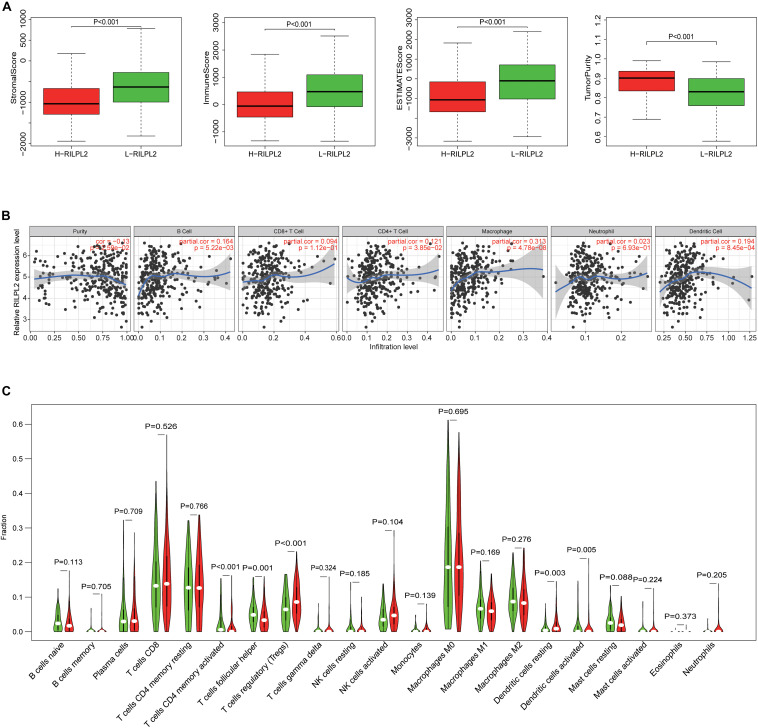
The correlation of TIICs infiltration with RILPL2 expression. **(A)** Comparison of stromal scores, immune scores, ESTIMATE scores, and tumor purity between the L-RILPL2 and H-RILPL2 groups according to the ESTIMATE tool. **(B)** TIMER analysis of the correlation between the expression of RILPL2 and six immune cells in EC. **(C)** The proportion of 22 immune cells in EC tissues in the L-RILPL2 and H-RILPL2 groups.

Based on the median expression value of RILPL2, 552 EC specimens obtained from the TCGA database were classified into high and low expression cohorts (H-RILPL2 and L-RILPL2 groups). It indicated that the L-RILPL2 group had a higher immune score and stromal score than the H-RILPL2 group *via* ESTIMATE analysis, while the tumor purity score was inferior (Figure 4A).

The TIMER analysis exhibited that RILPL2 expression had remarkablely positive correlations with B cells, CD4 + T cells, macrophages, and DCs in EC (Figure 4B). Moreover, we evaluated the correlations between RILPL2 expression and immune markers of different TIICs subtypes in EC tissues using TIMER database. The analysis exhibited that RILPL2 expression were correlated with the expression of marker genes of CD8 + T cells, T cells (general), B cells, monocyte, TAMs, M2 macrophage, neutrophils, NK cells, DCs, Th1 cells, Th2 cells, Th17 cells, Treg cells, and T cell exhaustion to varying degrees. Whereas, It revealed unrelated relationship between the expression of RILPL2 and the expression of gene markers for M1 macrophage and Tfh cell in EC samples (Table 2).

**TABLE 2:** Correlation analysis between RILPL2 and related gene markers of immune cells.

**Description**	**Gene markers**	***R* value**	***P* value**
CD8+ T cell	CD8A	0.141	0.001
	CD8B	0.105	0.014
T cell (general)	CD3D	0.211	<0.001
	CD3E	0.198	<0.001
	CD2	0.193	<0.001
B cell	CD19	0.246	<0.001
	CD79A	0.157	<0.001
Monocyte	CD86	0.247	<0.001
	CSF1R	0.319	<0.001
TAM	CCL2	0.135	0.002
	CD68	0.157	<0.001
	IL10	−0.111	0.010
M1 macrophage	NOS2	−0.021	0.631
	IRF5	0.025	0.562
	PTGS2	0.072	0.092
M2 macrophage	CD163	0.128	0.003
	VSIG4	0.213	<0.001
	MS4A4A	0.215	<0.001
Neutrophils	CEACAM8	0.005	0.915
	ITGAM	0.294	<0.001
	CCR7	0.182	<0.001
Natural killer cell	KIR2DL1	0.071	0.096
	KIR2DL3	0.066	0.125
	KIR2DL4	0.196	<0.001
	KIR3DL1	0.022	0.615
	KIR3DL2	0.035	0.419
	KIR3DL3	0.079	0.066
	KIR2DS4	0.028	0.517
Dendritic cell	HLA-DPB1	0.325	<0.001
	HLA-DQB1	0.218	<0.001
	HLA-DRA	0.333	<0.001
	HLA-DPA1	0.301	<0.001
	CD1C	0.419	<0.001
	NRP1	0.288	<0.001
	ITGAX	0.262	<0.001
Th1 cell	TBX21	0.155	<0.001
	STAT4	0.150	<0.001
	STAT1	−0.053	0.214
	IFNG	0.026	0.540
	TNF	0.028	0.516
Th2 cell	GATA3	−0.032	0.452
	STAT6	0.208	<0.001
	STAT5A	0.271	<0.001
	IL13	−0.020	0.641
Tfh cell	BCL6	0.024	0.582
	IL21	0.012	0.773
Th17 cell	STAT3	0.200	<0.001
	IL17A	0.013	0.758
Treg cell	FOXP3	0.119	0.005
	CCR8	0.086	0.046
	STAT5B	0.141	0.001
	TGFB1	0.032	0.453
T cell exhaustion	PDCD1	−0.010	0.808
	CTLA4	0.169	<0.001
	LAG3	−0.041	0.334
	HAVCR2	0.244	<0.001
	GZMB	−0.032	0.454

To distinguish the variance of the distribution of 22 TIICs between the two groups, CIBERSORT algorithm was applied to analyze EC cases from the TCGA database. The violin plot manifested the ratio differentiation of 22 TICs between EC tumor specimens with L- or H- RILPL2 expression. T cells CD4 memory activated, T cells follicular helper, T cells regulatory, Dendritic cells resting, and Dendritic cells activated were primary immune cells having a significant relationship with RILPL2 expression (Figure 4C). The proportion of all these five immune cells was increased in H-RILPL2 group compared with L-RILPL2 group.

### Methylation and Genetic Alterations of RILPL2 in EC

**FIGURE 5:**
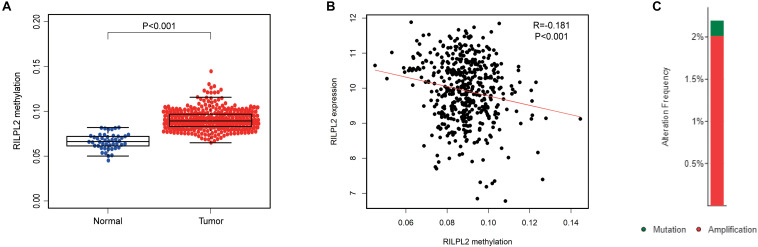
Methylation and mutation analysis of RILPL2 in EC. **(A)** Methylation level of RILPL2 in EC from the TCGA dataset. **(B)** Correlation between RILPL2 methylation level and its expression in EC. **(C)** The variety and proportion of samples with genetic alterations of RILPL2 in EC.

The DNA methylation level of RILPL2 was obtained from the TCGA database, and the differentially expressed methylation levels of RILPL2 between EC and para-cancerous specimens were analyzed. The methylation level of RILPL2 in the normal samples was notably lower than that in the tumor samples (*P* < 0.001, Figure 5A). Besides, the total methylation level of RILPL2 gene and methylation level of each site all showed significantly negative correlation with its expression (*P* < 0.001, Figure 5B and Supplementary Figure 1), which indicated that high methylation level was a significant cause for RILPL2 low expression in EC. Besides, low amplification and mutation rates of RILPL2 were found in EC patients (Figure 5C). Thus, genetic alterations might not be crucial for the dysregulation of RILPL2 in EC.

### Analysis of the Potential Mechanisms of RILPL2

**FIGURE 6:**
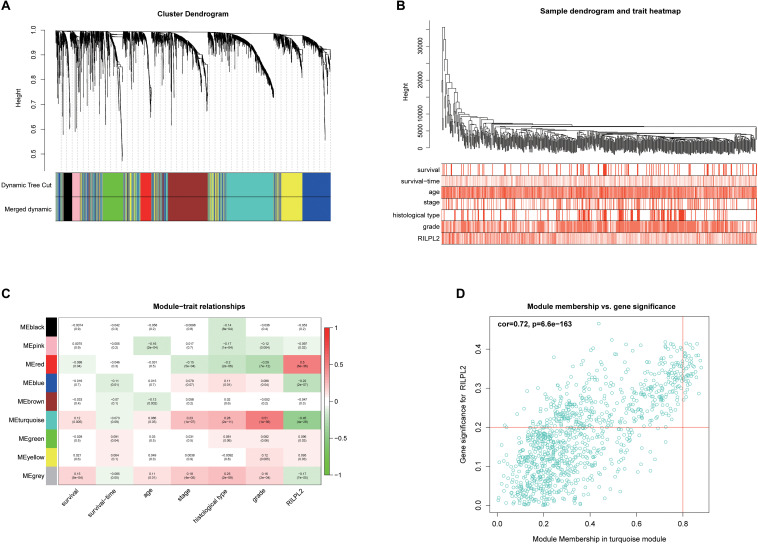
Exploration for genes correlated with RILPL2 in EC. **(A)** Clustering dendrogram of EC patients from the TCGA dataset. **(B)** A total of 2,573 DEGs were clustered according to the dissimilarity measure (1-TOM) and were classified into nine modules. **(C)** A correlation heatmap between module eigengenes and clinical features of EC. **(D)** Scatter plot of turquoise module eigengenes.

In order to build a weighted co-expression network and identify modules and genes related to RILPL2, 2,573 DEGs between EC and para-cancerous tissues from the TCGA database were submitted to WGCNA. After a series of adjustments for WGCNA parameters, the DEGs were clarified into nine modules by average linkage hierarchical clustering (Supplementary Figures 2A,B, Figures 6A,B). Among these modules, the turquoise module hinted the highest negative correlation with RILPL2 expression (Cor = −0.45, *P* < 0.001) (Figure 6C), which might indicated the RILPL2-involved potential oncogenic mechanisms. Subsequently, forty-two genes in the turquoise module were reserved as key genes (GS > 0.2 and MM > 0.8) (Figure 6D).

**FIGURE 7:**
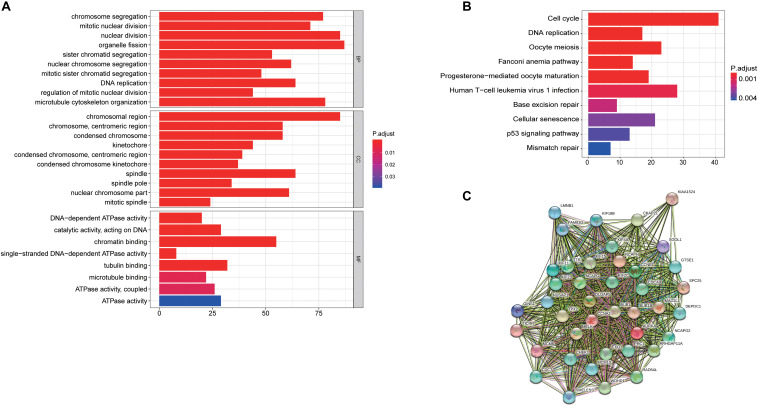
Latent oncogenic mechanisms of RILPL2 in EC. **(A)** GO analysis of genes in turquoise module eigengenes, top 10 terms were exhibited. **(B)** KEGG analysis of gene in turquoise module eigengenes, top 10 terms were exhibited. **(C)** PPIs of hub genes constructed by STRING tool.

To further understand the potential oncogenic mechanisms associated to these hub genes, we next conduct GO and KEGG analysis to perform. “chromosome segregation,” “chromosome region,” and “DNA-dependent ATPase activity” were the significantly important GO terms for cellular components, biological processes and molecular functions, respectively (Figure 7A). “cell cycle” was the most significant pathway in the KEGG pathway analysis (Figure 7B). Besides, we established a PPI network with these hub genes and found that a large numbers of cell cycle related genes play key roles (Figure 7C).

## Discussion

Endometrial carcinoma is one of the most widespread malignant tumors in female’s genital tract in the world and accounts for approximately 74,000 deaths per year worldwide (Ferlay et al., 2010; Siegel et al., 2017). The incidence of EC is increasing in the range of 10 to 20 per 100,000 women annually, and the age of onset is younger than in prior years (Moore and Brewer, 2017). The combination of surgery, radiotherapy, and chemotherapy is the current gold standards for treatment of EC patients. However, the 5-year survival rate is approximately 45–60% and 15–25% for stage III and stage IV EC, respectively, which led to the majority of EC-related deaths (Amant et al., 2005). Hence, it is of critical importance to identify credible biomarkers for early diagnosis, early initiation of treatments and improved prognosis of EC.

In this work, sequential data filtering was performed from the TCGA database, which determined the identification of the key gene RILPL2. The relationship between RILPL2 expression and clinical factors was analyzed by logistic regression analysis and clinical relevance filtering. Besides, we used Kaplan-Meier analysis, as well as univariate and multivariate Cox analysis to assess the associations between RILPL2 expression and survival outcomes. Not only that, we analyzed the relevance of RILPL2 expression with immune cells infiltration. Finally, the WGCNA analysis was conducted to perform the underlying mechanisms of RILPL2.

Previous research indicated that RILPL2 widely expressed in most tissues, including brain, heart, lung, liver, kidney, pancreas, and placenta (Wang et al., 2004). To our best knowledge, no available studies have revealed the expression of RILPL2 and its underlying prognostic impact in EC. However, relative studies indicated that RILPL2 showed a significant correlation with breast cancer by conducting series of literatures of RILPL2. Chen et al. revealed that RILPL2 expression in breast cancer tissues is lower than that in para-cancerous tissues, and that RILPL2 upregulation is associated with prolonged prognosis. Overexpression of RILPL2 inhibited breast cancer cell proliferation and metastasis *in vitro* and *in vivo*. Besides, the interaction of exogenous RILPL2 with TUBB3 resulted in the downregulation of breast cancer cell proliferation and migration and upregulation of PTEN expression by promoting destabilization of TUBB3 (Chen et al., 2019).

The present study focused on the potential role of RILPL2 in EC. We identified that RILPL2 was expressed differently in normal tissues and EC tissues. Kaplan-Meier analysis exhibited that high level of RILPL2 was notably related to better prognosis in patients with EC. From the result of univariate and multivariate Cox analysis, we found that RILPL2 was a high-risk factor and could be served as an independent indicator to forecast the clinical outcome of patients with EC. Besides, The ESTIMATE, TIMER, and CIBERSORT analysis both suggested RILPL2 might play a critical role in regulating immune cell infiltration in EC and is related to immune response. The fraction of immune cell infiltration plays crucial roles in tumor growth, metastasis, and therapeutic resistance (Waniczek et al., 2017). For example, CD8 + cytotoxic T cell serves as crucial role to destroy tumor cells in many cancers (Joyce and Fearon, 2015). Macrophages are classified into two classifications with various specific functions: M1, with a proinflammatory function, and M2, with an anti-inflammatory or wound healing effect (Demagny et al., 2014; Murray et al., 2014). Recent years, bioinformatics-dependent identification of immune-related biomarkers has been emerging, and increasing numbers of genes are found to be associated with tumor immunity (He et al., 2020; Mei et al., 2020). Our current research suggested that the expression of RILPL2 may regulate immune infiltration of multiple TIICs in the immune microenvironment of EC specimens, thus directly and/or indirectly regulating immune monitor and influencing progression of tumor.

## Conclusion

In summary, accumulating evidence on the tumorigenesis and development of RILPL2 in EC were acknowledged and preliminarily presents its potential as a promising diagnostic and prognostic indicator. Our results suggest that RILPL2 is decreased in EC and low RILPL2 expression is related to grade of malignancy and poor prognosis. In addition, our research also indicates that RILPL2 is closely correlated with immune infiltration in EC. Overall, these findings provide a theoretical basis for next underlying studies to validate the function of RILPL2 in EC.

## Data Availability Statement

The datasets presented in this study can be found in online repositories. The names of the repository/repositories and accession number(s) can be found in the article/Supplementary Material.

## Ethics Statement

The studies involving human participants were reviewed and approved by Nantong Third People’s Hospital Affiliated to Nantong University in 2020. The patients/participants provided their written informed consent to participate in this study. The animal study was reviewed and approved by Nantong Third People’s Hospital Affiliated to Nantong University in 2020.

## Author Contributions

HZ conceived the study and participated in the study design, performance, and manuscript writing. JHL, MX, ZW, and JQL conducted the bioinformatics analysis. HZ, YY, and SY revised the manuscript. All authors read and approved the final manuscript.

## Conflict of Interest

The authors declare that the research was conducted in the absence of any commercial or financial relationships that could be construed as a potential conflict of interest.

## Footnotes

1 https://portal.gdc.cancer.gov/

2 www.ncbi.nlm.nih.gov/geo

3 https://cistrome.shinyapps.io/timer/

4 http://cibersort.stanford.edu/

5 https://david-d.ncifcrf.gov/

6 https://string-db.org/

## Abbreviations

CDR: clinical data resource
DCs: dendritic cells
DEGs: differentially expressed genes
DFS: disease-free survival
DSS: disease-specific survival
EC: endometrial carcinoma
GEO: gene expression omnibus
GO: gene ontology
GS: gene significance
HCV: Hepatitis C virus
TCGA: the Cancer Genome Atlas
HPA: human protein atlas
KEGG: kyoto encyclopedia of genes and genomes
ME: module eigengene
MM: module membership
MS: module significance
OS: overall survival
PPI: protein-protein interaction
qRT-PCR: real-time quantitative RT-PCR
RILP: Rab-interacting lysosomal protein
RILPL2: Rab interacting lysosomal protein like 2
TIICs: tumor-infiltrating immune cells
TOM: top logical overlap matrix
WGCNA: weighted gene co-expression network analysis.
