# Supplementary material for: Low Expression of RILPL2 Predicts Poor Prognosis and Correlates With Immune Infiltration in Endometrial Carcinoma
Source: Front Mol Biosci. 2021 May 19;8:670893. doi: 10.3389/fmolb.2021.670893 (PMC8171931; doi:10.3389/fmolb.2021.670893)
Supplement: Supplementary file 1 [file Table_2.DOCX]

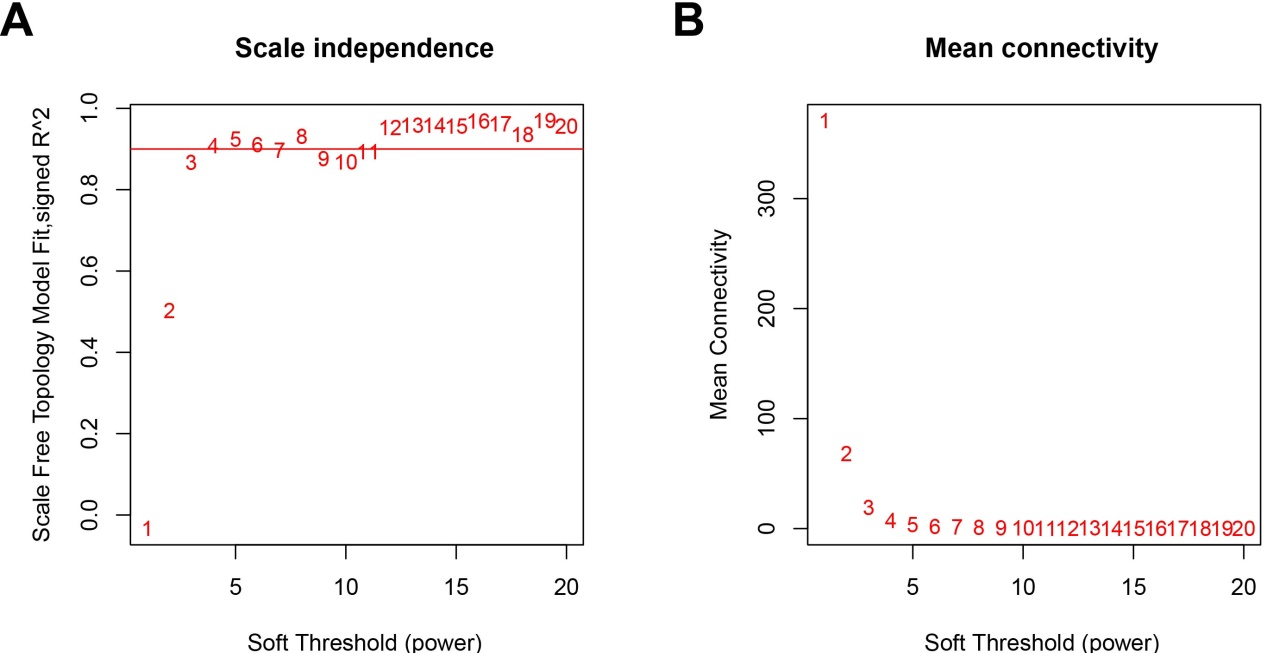


Figure S2. Adjustments for WGCNA parameters. (A) Calculation of the scale-free fit index of various softthresholding powers (β). (B) Analysis of the mean connectivity of various soft-thresholding powers (β).
