# Supplementary figures and images for: Low Expression of RILPL2 Predicts Poor Prognosis and Correlates With Immune Infiltration in Endometrial Carcinoma
Source: Front Mol Biosci. 2021 May 19;8:670893. doi: 10.3389/fmolb.2021.670893 (PMC8171931; doi:10.3389/fmolb.2021.670893)

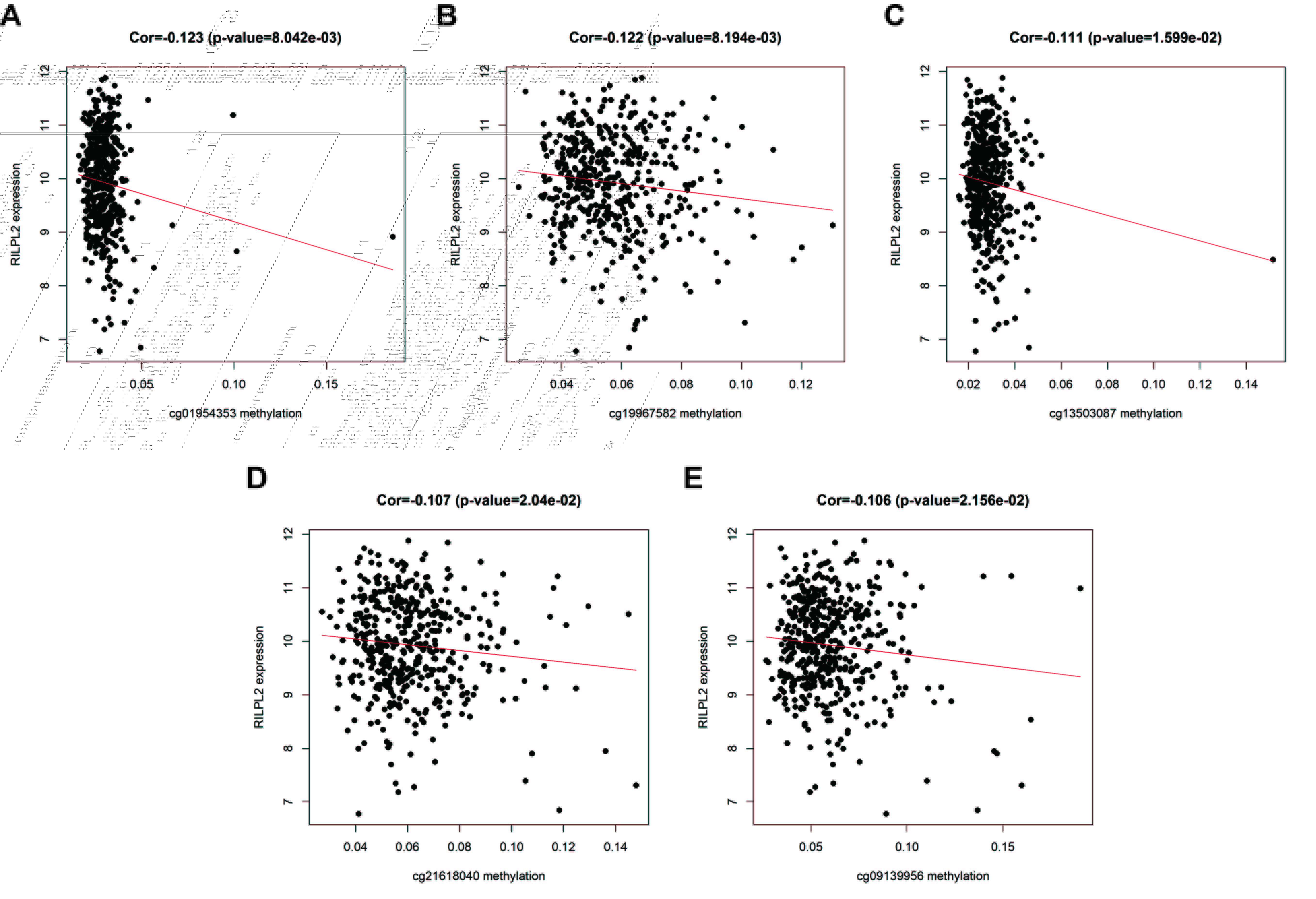

Supplement: Supplementary file 2 [file Image_1.jpg]
